# Calcarea-carbonica

**Published:** 1897-09

**Authors:** 


### HOMŒOPATHIC PHYSICIAN,

A MONTHLY JOURNAL OF

HOMŒOPATHIC MATERIA MEDICA AND CLINICAL MEDICINE.

"If our school ever gives up the strict inductive method of Hahnemann, we are lost, and deserve only to be mentioned as a caricature in the history of medicine."—CONSTANTINE HERING.

Vol. XVII. SEPTEMBER, 1897. No. 9.

### EDITORIAL.

CALCAREA-CARBONICA.—Calcarea has sour stool. This is Dr. Guernsey's keynote for Calcarea. Silicea has offensive diarrhœa. These symptoms occur especially in infants, and are the differential indications between Calcarea and Silicea, without regard to the appearance of the stool.

Calcarea has bloody urine, and so have some other remedies, especially Pulsatilla and Thuja. It also has hemorrhage from the urethra. The Calcarea patient is much troubled with micturition at night. This is similar to Lycopodium. The Calcarea urine sometimes smells sour.

Frequent nocturnal emissions indicate Calcarea, Silicea, Cobalt.

Calcarea has impotence with increased sexual desire, while Lycopodium has impotence without.

Calcarea has menses too soon, too long, and too profuse. This is Dr. Guernsey's keynote to Calcarea. Before the menses there is pain in the breasts, under Calcarea. It is similar to Kali-carb. and Murex.

Calcarea is a prominent remedy for hemorrhage of the uterus.

Calcarea is indicated in suppressed menses, especially with

"full habit." Kali-carbonicum has suppression of menses with anasarca.

Calcarea has griping pains in the back during menses. Kali-carb. has griping pains in back and bearing down in the abdomen.

Under Calcarea the mammary glands are hard and swollen.

Calcarea is an important remedy in leucorrhœa, especially is occurring before the menses.

Calcarea must be thought of in after pains and milk fever; also in galactorrhœa.

Calcarea has painless hoarseness. This is characteristic.

Calcarea has a cough which is worse at night during sleep.

Guernsey's keynote reads: "The child takes cold, a swelling comes in the neck, and there is oppression of the chest and cough."

Calcarea expectoration comes in the morning and during the day, rather than at night.

Calcarea is to be thought of in the ulceration of the lungs that affects stone-cutters.

Calcarea has tightness of the chest, as if too full of blood. This is similar to Belladonna.

Calcarea has continual desire to draw a deep breath. Lachesis also has this symptom.

Calcarea has sensitiveness and sensation of soreness of the chest when drawing a deep breath, and when touching it. The Editor can testify to the value of this symptom in treating people whose lungs are predisposed to tuberculosis. Especially is it true of the sensitiveness of the surface of the chest to touch.

Dr. Lippe's other notes are Stannum, soreness of chest, with much expectoration of white or green mucus tasting salt, especially in the morning.

Bryonia has soreness of chest, better from leaning against the painful side.

Galactorrhœa indicates both Calcarea and Rhus-tox.

Calcarea has pain in the small of the back, and so also have Lycopodium and Rhus-tox.

Calcarea has pressing pain between the shoulder-blades, which is similar to Silicea.

Curvature of the dorsal vertebræ suggests Calcarea and Silicea also.

Calcarea has tingling of the fingers, like Aconite. Paralysis of the arms suggests Calcarea. Lycopodium has paralysis of the left arm.

Calcarea has sudden weakness of the arms, as if from paralysis.

Nodes in the finger-joints suggest Calcarea and Petroleum.

Calcarea has sense of deadness of the fingers when grasping anything.

Calcarea also has perspiration of the hands.

Ipecac. has cold perspiration of the hands dripping from them.

Carbo-veg. has cold perspiration of tips of the fingers.

Lycopodium has perspiration of hands affected with gout to the extent of several drops a minute.

Lithium-carb., perspiration of back of hand while the palm is dry. This symptom was picked by the Editor out of Hering's *Guiding Symptoms.*

Calcarea is indicated in complaints of the patella.

Calcarea is a great remedy for cramps in the calves of the legs. These cramps are also successfully treated with Secale, Sulphur, and Veratrum, especially when the cramps extend to the soles of the feet and the toes.

This reminds the Editor of a case he treated of violent colic from eating clams out of season. There were reflex cramps of the calves, soles of the feet, and toes; both great toes standing perfectly vertically, and accompanied with the most agonizing pains, which caused the patient to scream. The attack had continued two hours before we were summoned, but immediately on our arrival we found that Arsenic was indicated, and we gave it without much delay, and in twenty minutes the pain ceased, the cramps were relieved, and the toes became perfectly flexible and assumed their natural position. This case is instructive in showing the need of having correlative symptoms to select the right remedy for these cramps of the extremities.

Calcarea is frequently indicated in phlegmasia alba. So also are indicated Apis, Bell., and Sulph.

Calcarea has burning of the soles of the feet like Sulphur.

Calcarea is indicated in foot sweat, the perspiration having a putrid smell. Silicea is the great remedy for this condition, and the Editor has had excellent success in prescribing it. A valuable repertory of foot sweat was published in The Homœopathic Physician for June, 1894.

Calcarea has coldness and deadness of the feet at night in bed. This is a characteristic.

Calcarea has great weakness and debility from a short walk, and even from talking. Kali-bichrom. has this symptom.

The Calcarea patient is sensitive to moist air.

Calcarea is a useful remedy in epileptic attacks, with unconsciousness; so has Silicea.

Nitric-acid has epilepsy with full consciousness. The Editor may remark that he has a case of epilepsy under treatment where there is unconsciousness during the height of the spasm, and yet he gives Nitric-acid with good results, the spasms having been reduced to one a year. Of course there were other symptoms that induced him to make this prescription.

